# Recent Developments at SSRL BL4-2, a Beamline for Biological Small and Wide Angle X-ray Scattering

**DOI:** 10.1063/4.0001162

**Published:** 2025-10-27

**Authors:** Thomas M Weiss

**Affiliations:** SSRL, Menlo Park, CA, USA

## Abstract

The small-angle x-ray scattering beam line BL4-2 at the Stanford Synchrotron Radiation Lightsource (SSRL) provides state-of-the-art experimental facilities for structural biology and biophysics research. The SAXS camera setup features a variable sample to detector distance (0.3 – 3.5m) and a Pilatus3 X 1M detector. Optionally an Eiger2 X 1M detector can be added for simultaneous small- and wide-angle scattering. A wide variety of specialized sample handling devices for variety of different SAXS experiments are available for use at the beam line. This includes a fully automated, remotely accessible, high-throughput solution sample delivery system, which performs all steps necessary for high quality data collection and is connected to a software pipeline for data reduction and initial analysis in real time. A size-exclusion chromatography setup can be directly coupled to the instrument to provide the highest sample quality SEC-SAXS data collection. This can also be further supplemented with inline MALS/DLS and RI detectors to increase the biophysical characterization of the sample. The customized data acquisition system provides fully automated SEC-SAXS data collection with real-time processing and display. Time-resolved solution SAXS on the millisecond time scale is also available at the beamline using and highly optimized stopped-flow system with 30ul minimal injection volume. For highly viscous and solid samples a sample holder system is available that holds up to 96 samples per cassette. A robotic system is available for changing the cassettes automatically or remotely providing prolonged high-throughput experimental capabilities. Recent technological developments and scientific results from the beamline will be presented.

